# A Novel Method for Proximity Detection of Moving Targets Using a Large-Scale Planar Capacitive Sensor System

**DOI:** 10.3390/s16050699

**Published:** 2016-05-16

**Authors:** Yong Ye, Jiahao Deng, Sanmin Shen, Zhuo Hou, Yuting Liu

**Affiliations:** 1 School of Mechatronical Engineering, Beijing Institute of Technology, Beijing 100081, China; bitdjh@bit.edu.cn (J.D.); shen_san_min@163.com (S.S.); houtso@163.com (Z.H.); liuyuting0531@126.com (Y.L.); 2 Science and Technology on Electromechanical dynamic Control Laboratory, Beijing Institute of Technology, Beijing 100000, China; 3 National Key Laboratory of Electronic Measurement Technology, North University of China, Taiyuan 030051, China

**Keywords:** moving target, proximity detection, planar capacitive sensor, sensitivity distribution

## Abstract

A novel method for proximity detection of moving targets (with high dielectric constants) using a large-scale (the size of each sensor is 31 cm × 19 cm) planar capacitive sensor system (PCSS) is proposed. The capacitive variation with distance is derived, and a pair of electrodes in a planar capacitive sensor unit (PCSU) with a spiral shape is found to have better performance on sensitivity distribution homogeneity and dynamic range than three other shapes (comb shape, rectangular shape, and circular shape). A driving excitation circuit with a Clapp oscillator is proposed, and a capacitance measuring circuit with sensitivity of 0.21 $V_{p-p}/\text{pF}$ is designed. The results of static experiments and dynamic experiments demonstrate that the voltage curves of static experiments are similar to those of dynamic experiments; therefore, the static data can be used to simulate the dynamic curves. The dynamic range of proximity detection for three projectiles is up to 60 cm, and the results of the following static experiments show that the PCSU with four neighboring units has the highest sensitivity (the sensitivities of other units are at least 4% lower); when the attack angle decreases, the intensity of sensor signal increases. This proposed method leads to the design of a feasible moving target detector with simple structure and low cost, which can be applied in the interception system.

## 1. Introduction

Planar capacitive sensors, whose sensors’ electrodes are placed in a coplanar plane [1], are widely applied to the measurement of various physical quantities such as humidity [2,3], size [4,5], speed [4], dielectric permittivity [6], thickness [6,7], rain [8], moisture content [9], and voltage [10]. In addition, methods for the detecting of material cracks and material delaminations using planar capacitive imaging techniques [11] have been developed. The sensing modes of planar capacitive sensors, including the transmission mode, single-electrode mode and shunt mode, have been summarized by Hu *et al.* [1]: the transmission mode or single-electrode mode is suitable for material characterization and imaging [12], while the shunt mode is suitable for proximity/displacement measurement. However, the material under testing is grounded in the shunt mode, so it is not suitable for some applications of proximity measurement. On the other hand, in the transmission mode, the sensor output is correlated to the position of a material under testing, making this mode also suitable for proximity/displacement measurement. For instance, Kirchner *et al.* [13] used planar capacitive sensors in the transmission mode for object ranging and material type identification, and Eidenberger *et al.* [14] also used it for the measurement and tracking of edge angles. Additionally, Kao *et al.* [15] presented a non-invasive technique using planar electrode arrays for breast cancer diagnosis. They discussed the advantages and disadvantages of four manners of driving excitation: using a single voltage source, a single current source, a multiple current sources with a fixed pre-determined “canonical” pattern of currents and an adaptively determined ‘optimal’ pattern of currents. In this paper, a PCSS (planar capacitive sensor system) for proximity detection of moving targets (with high dielectric constant) is considered.

Currently, there are many moving-target detection techniques based on radar, infrared, laser, and magnetic sensor, *etc.*, but the applications of these techniques are restricted to various degrees by the long response time of sensors (such as radar), the influences of the environment (such as infrared and laser), and insensitivity of targets made of nonmagnetic material (such as magnetic sensor). Compared with other technologies, capacitive sensors exhibit nice properties: design simplicity, low power consumption, high sensitivity [11] and satisfactory stealth.

Our goal in this work is to develop a feasible, inexpensive and simple system of moving target proximity detection. At first, the detection principle is derived. Compared with existing planar capacitive sensors, this study presents large-size (areas up to hundreds of square centimeters) sensors for detection within a relatively long distance (detection distance more than 40 cm). For such a sensor plate, the front face of the moving target is relatively small, and the sensitivity distribution of a single planar sensor can influence the signal intensity. Thus, the sensitivity distributions of four electrode shapes are discussed in Section 3. Section 3 also presents designed circuits for driving excitation and capacitance measurement. Section 4 illustrates the results of experiments in details. Finally, Section 5 concludes the paper.

## 2. Detection Principle

**Figure 1:**
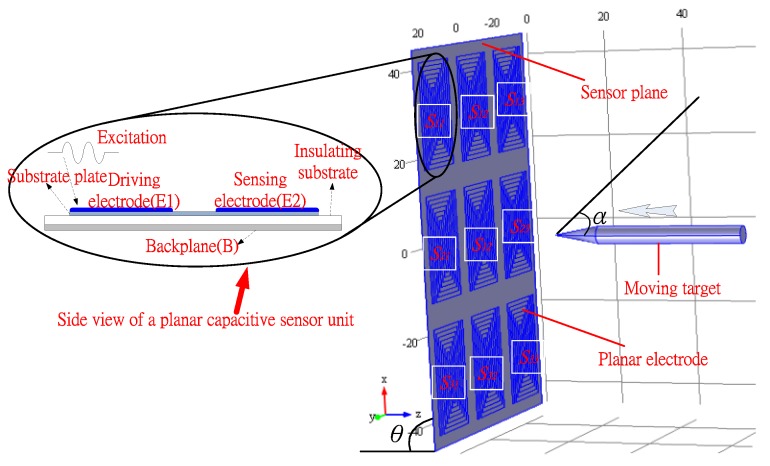
Schematic of the planar capacitive sensors system (PCSS) structure.

**Figure 2:**
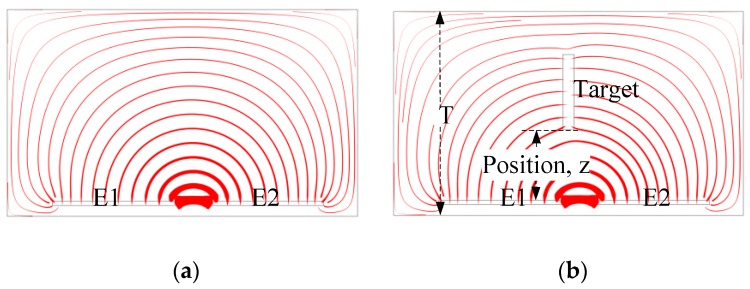
Schematic of electric field lines without a target (**a**) and with a target ($\varepsilon_{T}=52$) (**b**). The setting of the boundary conditions in the simulation are as follows: the dielectric of the electrodes and the target are 100 and 52, respectively; the electrical potential of the driving electrode and the sensing electrode are 5 and 0 V; the air boundary was defined as zero charge.

The proposed planar capacitive sensor system contains several planar capacitive sensor units (PCSU) which are arranged in arrays forming a matrix in a plane, as shown in Figure 1. The system can detect the variation of signals when a moving target comes close. The PCSU consists of a pair of coplanar electrodes (the driving electrode E1, and the sensing electrode E2), and a substrate plate (S). The planar electrodes are made by erosion on the substrate plate, and the planar capacitive sensor unit as a whole is placed on the insulating substrate and the backplane. The driving electrode is powered by an external voltage, and then a quasi-electrostatic field over the area of the coplanar electrodes is established. Generally, an electrostatic field is divided into two fields: the near-zone field and the radiated field. In order to obtain higher sensitivity, the PCSU work in the near-zone field and detect signals with oscillation frequencies in the range of kHz to MHz [16]. Capacitive sensing of moving targets in the detection area is based on the disturbance in the electric field caused by a dielectric, as shown in Figure 2a,b. Without a target, the electric field lines are drawn from the driving electrode toward the sensing electrode; the presence of a target counteracts the effect of the driving electrode on the sensing electrode.

**Figure 3:**
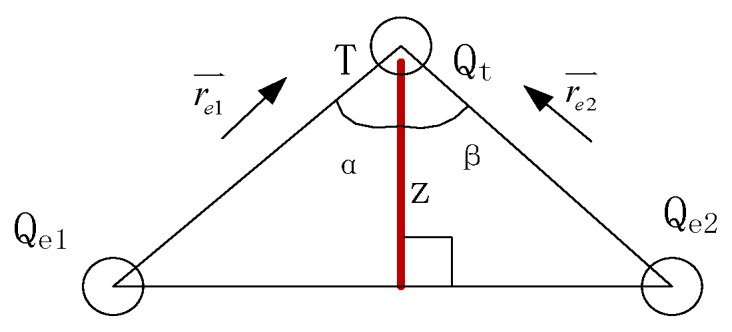
Schematic of electric field vector

To simplify theoretical analysis, the two electrodes and a target can be modeled as three particles. When the distance z (the distance from the electrode plane to the target face in the Z-direction) is less than *T* (a maximum field penetration depth), *i.e.*, z<T, the polarization charge $Q_{t}$ is generated on the target because of the electric fields of the driving electrode and sensing electrode. Hence, the contribution of electric fields to the polarization change can be written as:

(1) $$Q_{t}=\varepsilon_{0}\chi_{e}(\vec{E_{e1}}+\vec{E_{e2}})$$

where $\chi_{e}$ is the electric susceptibility of the target, which is related to the target’s relative permittivity $\varepsilon_{e}$: $\chi_{e}=\varepsilon_{e}-1$; $\vec{E_{e1}}$ and $\vec{E_{e2}}$ are the electric-field intensity at the spatial position of the target caused by the driving electrode and the sensing electrode, respectively, and they can be expressed as:

(2) $$\left\{ \begin{array}{l} \vec{E_{e1}}=-\nabla \phi_{e1}=\frac{Q_{e1}}{4\pi \varepsilon_{0}R_{e1}}\vec{r_{e1}}=\frac{Q_{e1}}{4\pi \varepsilon_{0}z/\sin\alpha }\vec{r_{e1}}\\ \vec{E_{e2}}=-\nabla \phi_{e2}=\frac{Q_{e2}}{4\pi \varepsilon_{0}R_{e2}}\vec{r_{e2}}=\frac{Q_{e2}}{4\pi \varepsilon_{0}z/\sin\beta }\vec{r_{e2}} \end{array}\right.$$

where $Q_{e1}$ and $Q_{e2}$ are the charges on the driving electrode and the sensing electrode, respectively, and α and β are defined as the attitude angles shown in Figure 3.

According to the law of the conservation of electric charges, the correlation among the three conductors can be written as:

(3) $$\left\{ \begin{array}{ll} Q_{e1}=-Q_{e2} & \text{without a target}\\ Q_{e1}=-(Q_{e2}+Q_{t}) & \text{with a target} \end{array}\right.$$

Hence, the variation of the charges on the sensing electrode during the approaching of a target can be calculated as:

(4) $$\Delta Q_{e2}=Q_{t}$$

Thus, combining Equations (1) and (2), $\Delta Q_{e2}$ can be rewritten as

(5) $$\Delta Q_{e2}=\frac{\varepsilon_{0}\chi_{e}}{4\pi \varepsilon_{0}z}(\sin\alpha Q_{e1}\vec{r_{e1}}+\sin\beta Q_{e2}\vec{r_{e2}})=f(\chi_{e},z,\alpha ,\beta )$$

**Figure 4:**
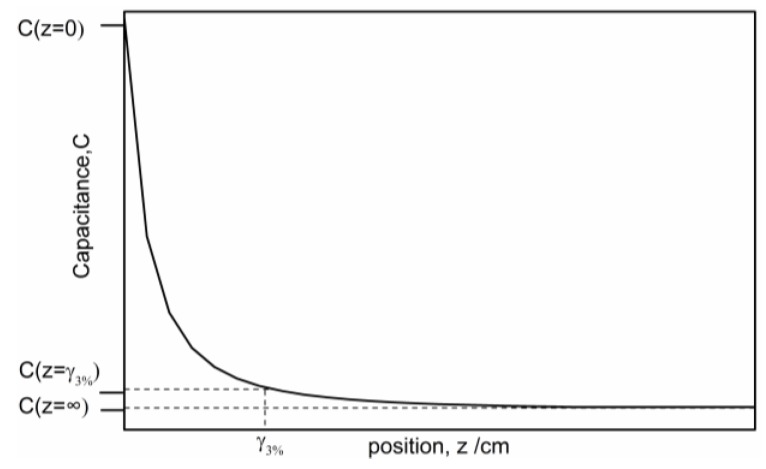
Analytical curve of ΔC-z in theory.

Then, the capacitance variation ΔC between the electrodes can be defined as:

(6) $$\Delta C=\frac{\Delta Q_{e2}}{V}=\frac{f(\chi_{e},z,\alpha ,\beta )}{V}$$

where V is the potential of the driving electrode. Because the driving electrode is the source, V can be regarded as a constant. Equation (6) shows that the correlation between the capacitance variation, and the distance is ΔC∝1/z. Figure 4 illustrates the capacitance-distance curve. The dynamic range, or the penetration depth, of a planar capacitive sensor can be defined as the position difference in the Z-direction between the position ($\gamma_{\;3\%}$) where 3% capacitance variation occurs and the position where the maximum capacitance change occurs [17], as shown in Figure 4. Hence, the variation of capacitance can be used to detect the moving target.

## 3. Sensor Design

In this section, theoretical analysis, numerical simulation and experiments are conducted for a number of sensor design issues: evaluation of the sensitivity distributions of different electrode shape designs, and the response time of the capacitive measuring circuit.

### 3.1. Sensitivity Distribution of Electrode Shape Design

In the applications of capacitive sensors, sensitivity distribution is a key issue, especially in an electrical capacitance tomography (ECT) system. The definition of sensitivity distribution has been given in the literature [18,19,20,21,22]:

(7) $$S_{i,j}(x,y)=-\int_{P(x,y)}\frac{E_{i}(x,y)}{V_{i}}.\frac{E_{j}(x,y)}{V_{j}}dxdy$$

where $E_{i}(x,y)$ and $E_{j}(x,y)$ are the intensity of electric field at the location (x,y) when the *i*th and *j*th electrodes are connected to voltages $V_{i}$ and $V_{j}$, respectively [20], and P(x,y) is the sensing space.

Different electrode shapes may also lead to different sensitivity distributions. In this study, four electrode shapes are designed: (a) circular shape, (b) rectangular shape, (c) spiral shape and (d) comb shape, as shown in Figure 5a–d, respectively. In order to compare the sensor sensitivity distributions of the four electrode shapes, the electrodes are placed in an approximately equal area in each design, *i.e.*, $S_{a}\approx S_{b}\approx S_{c}\approx S_{d}$ = 2 × π × 7^2^ mm^2^ = 307.72 mm^2^, and the spacings between electrodes are all 1 cm in the four electrode shapes.

The sensitivity distributions of the four electrode shapes are simulated with the finite element method (FEM) method using the software COMSOL Multiphysics (version 5.0, COMSOL Inc., Burlington, MA, USA). In each finite element (FE) model, the electric potentials of the driving electrode and the sensing electrode are set to be “5 V” and “Ground”; the volume of the computational domain is 35 cm × 20 cm × 10 cm; the mesh generation density is set to be “Extremely fine”; and the mesh number is set to be 385,740.

Figure 5a–d shows the results of simulation, in which large electric field variables are mainly distributed in the area of the gap between two electrodes. It is clearly shown that the spiral shape and comb shape have higher average levels of sensitivity distribution. This conclusion is also confirmed in the following experiment.

**Figure 6:**
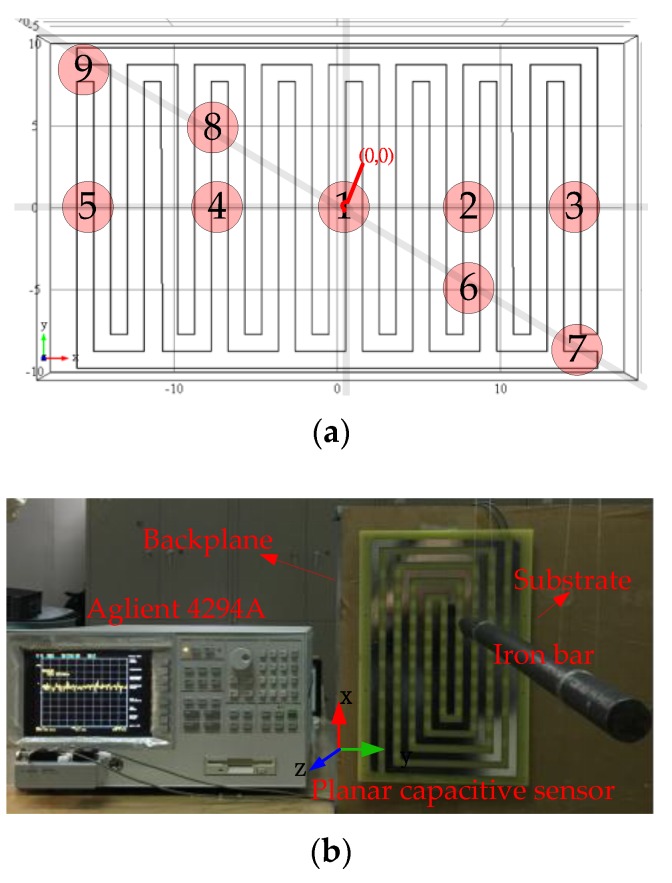
(**a**) Prototype of the spiral electrodes and (**b**) measurement experiment using the precision impedance analyzer (Aglient 4294A).

Figure 6b shows an experiment conducted with an Aglient 4294A precision impedance analyzer (Aglient Inc., Santa Clara, CA, USA). The measured object to detect is a static iron bar whose length is 430 mm and diameter is 22 mm. The four tested electrode shapes in the experiment have the same sizes as those in the simulation. The driving excitation frequency and amplitude are set to be 2.3 MHz and 1 V, respectively, and the Direct Current (DC) bias is set to be 4.5 V. The tested planar electrodes and substrate plate are made of tin and flamed FR-4, respectively. Because the four electrode shapes are symmetrical about the center, nine sensitivity positions in the electrode plane are selected as the testing positions, as shown in Figure 6a. The coordinates of the nine sensitivity positions 1#– Direct Current (DC)9# in the XY plane are (0,0), (8,0), (15,0), (−8,0), (−15,0), (8,−4), (15,−8), (−8,4), and (−15,8). The spacings of electrodes are all 1 cm in the four shapes. The axis of iron bar is parallel to the *z*-axis.

**Figure 7:**
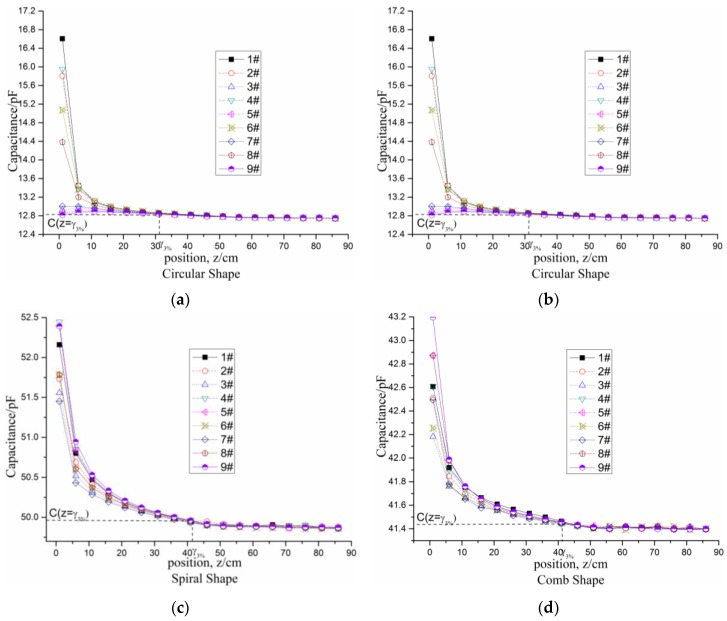
Experimental results of testing the four electrode shapes: (**a**) Circular shape; (**b**) Rectangular shape; (**c**) Spiral shape and (**d**) comb shape, from 1 cm to 86 cm at intervals of 5 cm.

The capacitance variations with the distance (from the electrode plane to the target front face) in the *z*-axis for the four electrode shapes are shown in Figure 7a–d. Using 1# sensitivity position as the reference sensitivity position, two parameters are defined to evaluate the similarity between the capacitance variation curve at 1# sensitivity position and those at other sensitivity positions: the correlation coefficient and residual standard deviation:

(8) $$\text{Correlation coefficient }=\frac{\sum_{j=1}^{N}\left(C_{i\#,j}-C_{1\#,j})(\overset{\wedge }{C_{i\#,j}}-\overset{\wedge }{C_{1\#,j}}\right)}{\sqrt{\sum_{j=1}^{N}{\left(C_{i\#,j}-C_{1\#,j}\right)}^{2}\sum_{j=1}^{N}{\left(\overset{\wedge }{C_{i\#,j}}-\overset{\wedge }{C_{1\#,j}}\right)}^{2}}}$$

(9) $$\text{Residual standard deviation }=\sqrt{\frac{\sum_{j=1}^{N}{\left(C_{i\#,j}-C_{1\#,j}\right)}^{2}}{N-2}}$$

In the two above equations, $C_{i\#,j}$ and $\overset{\wedge }{C_{i\#,j}}$, are the real capacitance and the estimated capacitance of the i# sensitivity position at the *j*th distance point. “N” is the measurement time, and the number “2” refers to the number of undetermined constants.

Figure 7a–d illustrate the experimental results of testing the four electrode shapes. The dynamic ranges of the four electrode shapes are approximately 31, 31, 41, and 41 cm, respectively.

**Figure 8:**
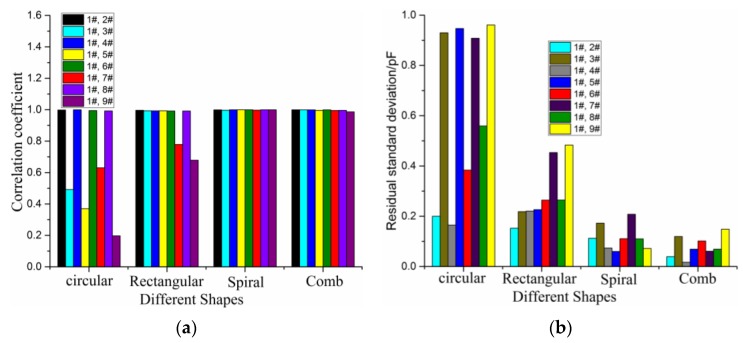
Correlation coefficient (**a**) and residual standard deviation (**b**) between 1# sensitivity position with others sensitivity positions, respectively, for the four electrode shapes.

Figure 8a,b shows the correlation coefficient and the average error between 1# sensitivity position and other sensitivity positions for the four electrode shapes. It is found that the curvilinear correlations (all correlation coefficients are more than 99.3%) of the spiral electrode shape and the comb electrode shape are higher than those of the other two electrode shapes. Meanwhile, the standard residual deviations (not exceeding 0.21 pF) of the spiral electrode shape and the comb electrode shape are the lowest. The signal intensity of the spiral shape is 2.53 pF more than that of the comb shape (1.80 pF). Therefore, the spiral shape has the best performance in terms of sensitivity distribution homogeneity, signal intensity and dynamic range, the next is the comb shape, the third is the rectangular shape, and the circular shape has the worst performance. In summary, the spiral shape is the optimal electrode geometry among the four shapes under consideration.

### 3.2. Driving Excitation and Capacitance Measuring Circuit

**Figure 9:**
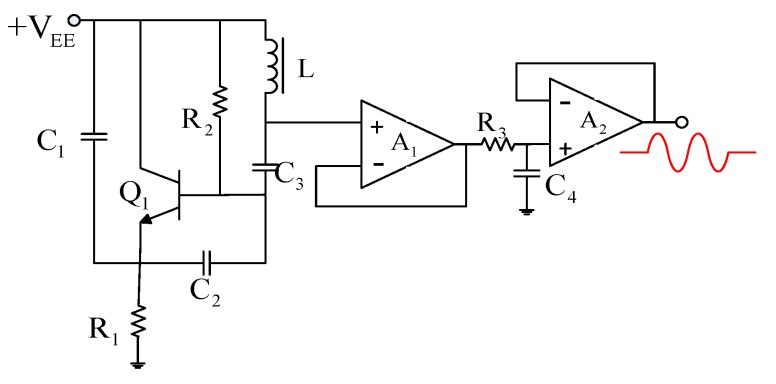
A Clapp oscillator as the driving excitation. The oscillation frequency is 2.3 MHz, and the oscillation amplitude is about 1 V.

A Clapp oscillator generating a sine signal as the driving excitation is shown in Figure 9. The Clapp oscillator has a large *C*_3_ to ensure high-frequency stability, hence a better choice for the driving excitation. In general, $C_{3}\ll C_{1}$ and $C_{3}\ll C_{2}$, and the oscillation frequency can be calculated as:

(10) $$f=\frac{1}{2\pi \sqrt{LC_{3}}}$$

When L and $C_{3}$ are selected to be 47 μH and 82 pF, respectively, the oscillation frequency is 2.3 MHz. Then, $R_{1}$, $R_{2}$, $C_{1}$, and $C_{2}$ are selected to be 20 kΩ, 1 kΩ, 470 pF and 1000 pF, respectively. In order to reduce the effect of the subsequent circuit on the Clapp oscillator, the amplifier $A_{1}$ is designed as a voltage follower circuit, and the sine signal is then filtered by a 1st-order low-pass filter. The oscillation amplitude is about 1 V, and the DC bias is about 4.5 V (the same as in the experiment introduced in Section 3.1).

**Figure 10:**
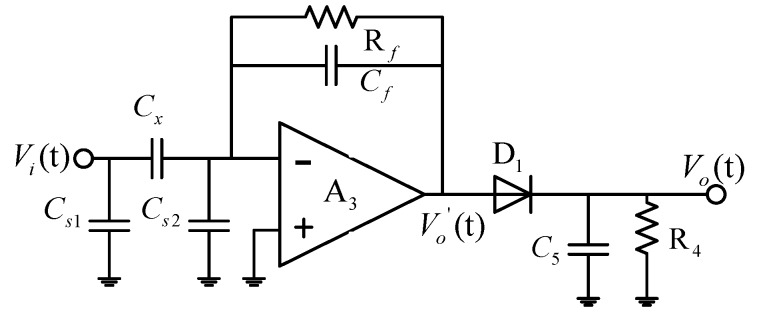
The C/V circuit.

To measure the capacitance, the C/V circuit and capacitance-to-digital converter (CDC) (chip AD7466 (Analog Devices, Inc., Norwood, MA, USA) *et al.*) circuit are widely used methods. Compared with the CDC circuit, the C/V circuit is more suitable for high speed measurement. The C/V circuit is shown in Figure 10, where $C_{x}$ is the measured capacitance, $C_{f}$ and $R_{f}$ are the feedback capacitance and feedback resistance, respectively, and $C_{s1}$ and $C_{s2}$ are the stray capacitances. They are all set to be 100 pF. The output voltage of the operational amplifier $A_{3}$ can be calculated as:

(11) $${V_{o}}^{\prime }(t)=-\frac{jwC_{x}R_{f}}{jwC_{f}R_{f}+1}V_{i}(t)$$

where ω is the angular frequency. When $\left|jwC_{f}R_{f}\right|\gg 1$, Equation (11) can be simplified as [22]:

(12) $${V_{o}}^{\prime }(t)=-\frac{C_{x}}{C_{f}}V_{i}(t)$$

According to the results measured using Aglient 4294A, the capacitance of the spiral electrodes is about 38.2 pF without the presence of a target in the laboratory environment, and the capacitance variation is in several pFs. Because $C_{f}$ and $R_{f}$ are selected to be 4.7 pF and 30 kΩ, respectively, the output voltage of the operational amplifier $A_{3}$ is about 10 $V_{p-p}$, so an ADA4610 is used as the operational amplifier $A_{3}$, which is powered by ±15 V DC. The response time is about 1 μs ($t_{sponse}=6.9C_{f}R_{f}$ [23]).

**Figure 11:**
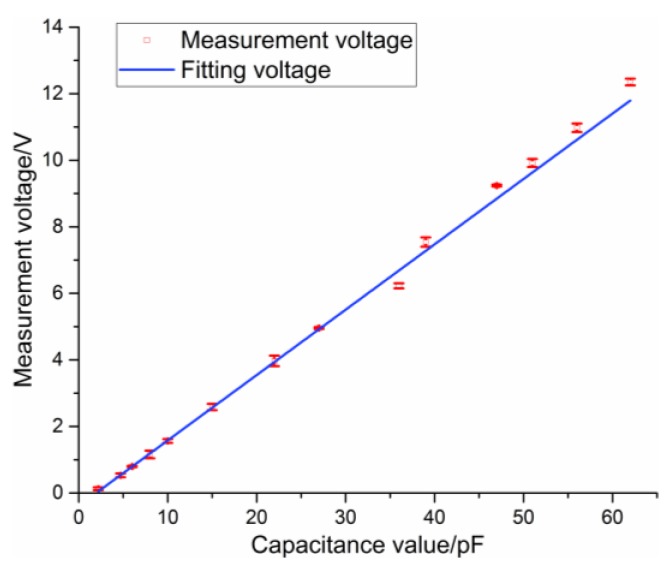
Input-output curve of the C/V circuit.

For the output of operational amplifier $A_{3}$, the amplification of signal is limited by the high-voltage limit of the amplifier. In this paper, a C/V circuit with a diode half-wave rectifier capacitor filter circuit is used. The C/V circuit converts the variation of capacitance to the variation of a DC signal. Generally, when “$C_{5}R_{4}\gg (3∼5)*2\pi /f$” is satisfied, the output signal becomes smooth and stable. When $C_{5}$ and $R_{4}$ are selected to be 10 nF and 1.5 kΩ, respectively, the response time is about 45 μs ($3C_{5}R_{4}=45\;\mu \text{s}$). The output of the half-wave rectifier can be expressed as:

(13) $${V_{o}}^{\prime }(t)=K{{V_{o}}^{\prime }}_{p-p}=-K\frac{C_{x}}{C_{f}}V_{p-p}$$

where K is a constant related to the load. The measurement results showed that K is about 0.87. Then, the sensitivity of the C/V circuit is 0.21 $V_{p-p}/\text{pF}$. The input–output curve of the C/V circuit is shown in Figure 11, which shows that the maximum absolute error and linearity error are 0.55 pF and 0.83%, respectively.

**Figure 12:**
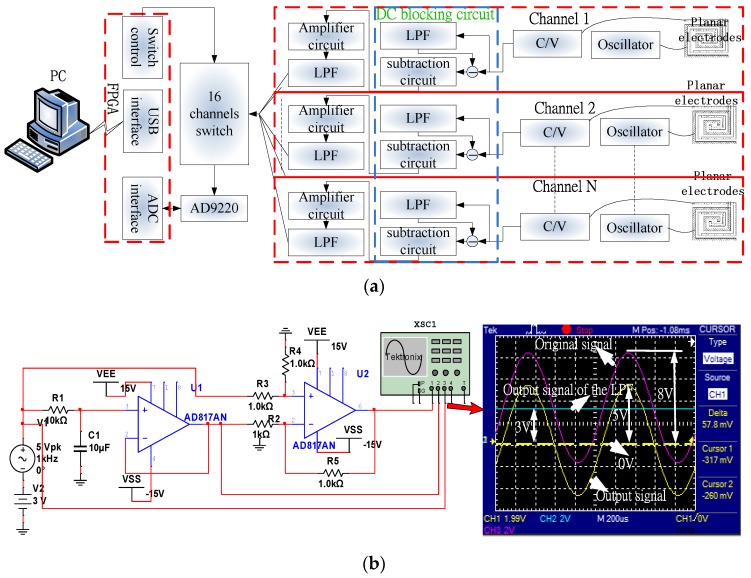
Illustration of (**a**) the digital capacitance measuring circuit and (**b**) the DC blocking circuit. The DC blocking circuit consists of an LPF and a subtraction circuit. The LPF is to filter AC signals by setting a lower cutoff frequency and is subtracted from the original signal. In a simulated experiment, the input was a 5 V sine signal of 3 V DC bias; the output of the LPF is 3 V DC voltage and the output of the subtraction circuit is a 5 V sine signal.

The digital circuit for measuring capacitance shown in Figure 12a has the function of multichannel (up to 16 channels) acquisition of the signals from capacitive sensors. It consists of a DC blocking circuit, an amplifier circuit, a low pass filter (LPF), a 16-channel switch, an Analog-to-Digital Converter (ADC), a field programmable gate array (FPGA), and a PC. The DC blocking circuit consists of an LPF and a subtraction circuit and works to block direct current signal and directly detect the voltage variation. The LPF of the DC blocking circuit serves to filter out alternating current (AC) signals with frequencies lower that a pre-set cutoff frequency, which are then subtracted from the original signal. Compared to the blocking capacitor method, this method has no output delay, as shown in Figure 12b. The response time of this digital capacitance measuring circuit depends primarily on the C/V circuit, and the post LPF and is about 60 μs in this study.

## 4. Experiments

### 4.1. Experiment Setup

In this study, a PCSS consisting of nine PCSUs (3 × 3 array) arranged with 5 cm parallel spacing is tested, as shown in Figure 1, and the electrodes have spiral shape. The size of the spiral electrodes is 31 cm × 19 cm. The planar electrodes and substrate plate are made of tin and flamed FR-4, respectively, and the planar capacitive sensor unit as a whole is placed on the insulating substrate. The insulating substrate and the backplane are made of plexiglass and iron, respectively. The thicknesses of the insulating substrate and the backplane are all 10 mm.

**Figure 13:**
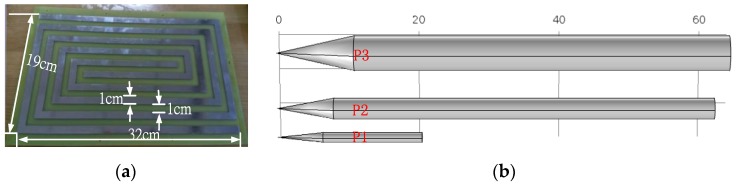
(**a**) Prototype of the spiral electrodes and (**b**) schematic view of three projectiles.

The PCSS is designed to detect the presence of a moving target (projectile). Taking into account the potential application of this study, three projectiles are selected as the targets for detection, as shown in Figure 13, and they are numbered P1–P3. The sizes of the three projectiles are shown in Table 1.

The same instruments used in Section 3.1 are used. The frequency of the driving excitation is 2.3 MHz, and the amplitude is about 1 V. The sensor plane is parallel to the *XY* plane, and the projectile axis is parallel to the *z*-axis.

There are three objectives in the following experiments: (1) comparison of the dynamic (the object under test is a moving target) and static (the object under test is a static target) experiments for the feasibility of moving-target proximity detection, (2) examining the sensitivity variations with different PCSUs, and (3) with the signals for targets moving at different attack angles.

In the dynamic experiments, the average speeds of 1048, 400 and 700 m/s are tested for the three projectiles. The projectile flight paths are measured by using a high-speed camera system with measurement speed at 21,000 fps. The sampling rate for each channel of capacitance measuring circuit is 191 kHz. The voltage signals are sampled using AD9220 (the input voltage range from 0 V to 5 V). To compare the data of dynamic and static experiments, the output signals of the C/V circuit are taken as the data of static experiment. All the sampled signals in the experiments are amplified by two times.

### 4.2. Experiment Results and Discussion

**Figure 14:**
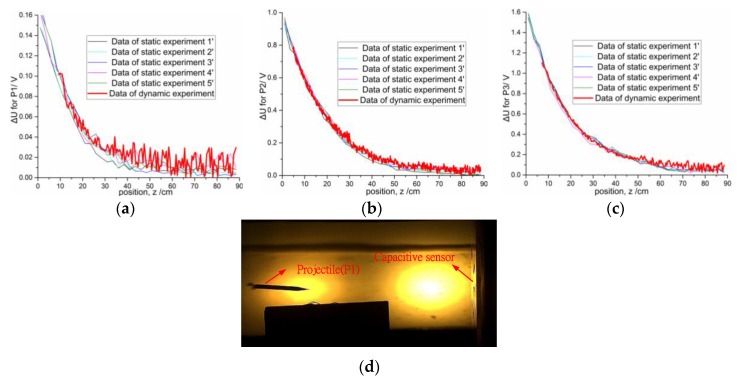
Comparisons of the static experiment data and the dynamic experiment data for three projectiles at the attack angle 90°: (**a**) P1; (**b**) P2; (**c**) P3; (**d**) Prototype of the dynamic experiment.

Figure 14a–c displays the voltage variation curves detected with a single capacitive sensor in the static and dynamic experiments for the three projectiles at the angle of attack of 90°. The static experiment is repeated five times for each projectile. The plots reveal that, under the same condition, the bigger the volume of the target, the larger the voltage variation and the larger the dynamic range. When the distance of the target changes from 1 cm to 88.5 cm, the variations of capacitance are about 360 fF, 2.27 pF and 3.63 pF for projectile P1, P2, and P3, respectively. The dynamic ranges are approximately 30, 46, and 57 cm for projectile P1, P2, and P3, respectively. The data of dynamic experiment is not recorded completely because the digital capacitance measuring circuit has time delay. Figure 14a–c shows that the voltage variation in the static experiment is similar to that in the dynamic experiment, suggesting that the results of static experiments could represent the behavior of the same projectile in the dynamic experiment.

**Figure 15:**
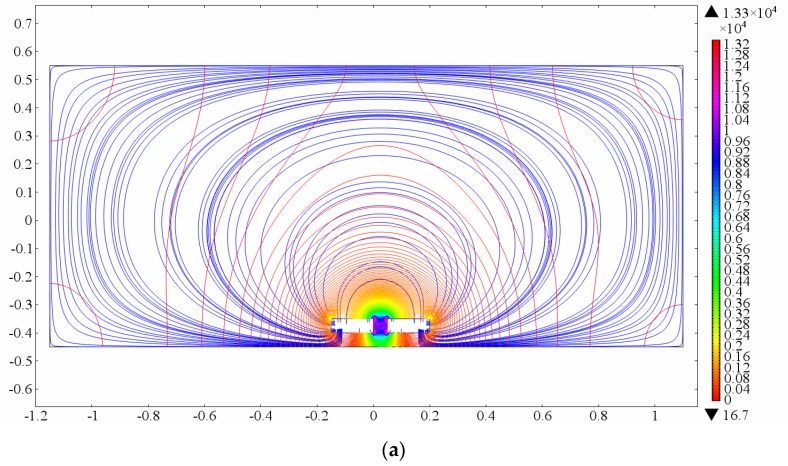

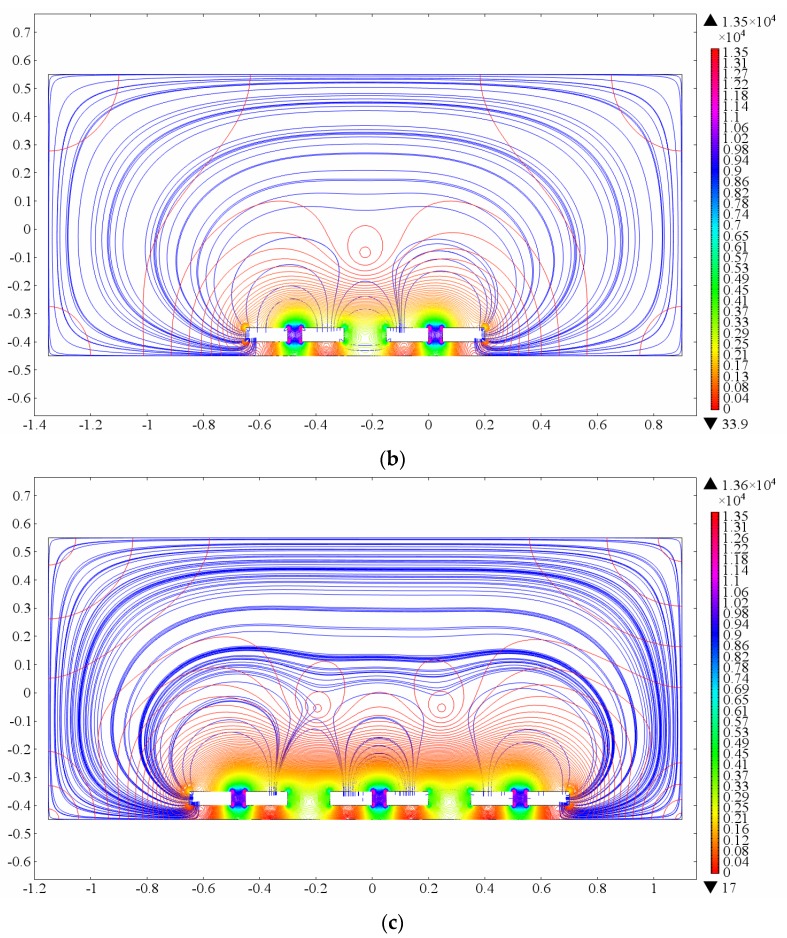
Schematic view of interaction between different number of PCSUs: a unit (**a**); two units (**b**); three units (**c**).

Crosstalk often resides among different PCSUs, especially the neighboring units. Crosstalk can bring two problems: noise increase, and change of signal intensity. Fabrication errors of IC in the Clapp oscillator are responsible for noise increase. Rigorous screening on IC to ensure parameter consistency is an effective way to reduce the noise level. The 2D simulation results shown in Figure 15a–c can illustrate the change of field lines and electric field with different numbers of PCSUs. As the number of PCSUs increase, the electric field lines interweave each other, and the electric field is enhanced in the sensing domains. The PCSU on the top left corner of the sensor array is named as $S_{11}$, and others are defined successively as $S_{12}$–$S_{33}$, as shown in Figure 2a. According to the number of neighboring units, $S_{11}$ (has two neighboring units), $S_{12}$ (has three neighboring units), and $S_{22}$ (has four neighboring units) types are selected as the measurement units for sensitivity comparison.

**Figure 16:**
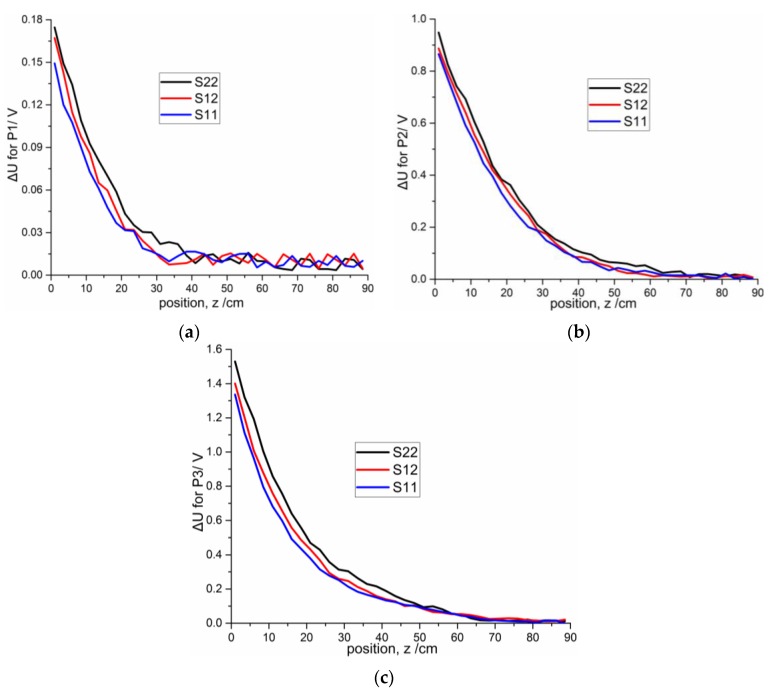
Comparisons of the static experiment data of $S_{11}$, $S_{12}$ and $S_{22}$ for three projectiles with distances from 1 cm to 88.5 cm at intervals of 2.5 cm: (**a**) P1; (**b**) P2; (**c**) P3.

Figure 16 shows the results of the static measurements using $S_{11}$, $S_{12}$ and $S_{22}$ for the three projectiles. The same trend exists in the static experiments results for the three projectiles: the voltage variation of $S_{22}$ is larger than those of $S_{12}$ and $S_{11}$. The voltage variations of $S_{11}$ and $S_{12}$ are 14.11% and 4.35% lower than $S_{22}$, respectively, for P1, 8.74% and 5.84% lower than $S_{22}$, respectively, for P2, and 12.57% and 8.37% lower than $S_{22}$, respectively, for P3.

**Figure 17:**
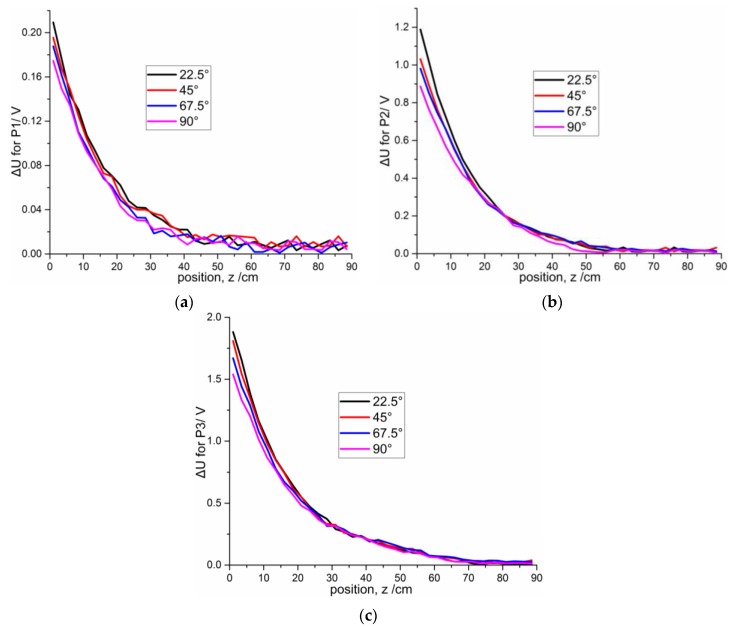
Comparisons of different attack angles for three projectiles with distance from 1 cm to 88.5 cm at intervals of 2.5 cm: (**a**) P1; (**b**) P2; (**c**) P3.

To investigate the influences of different attack angles on signal intensity variation, twelve independent experiments are performed. Figure 17a–c shows the variation of signal intensity with angle of attack (22.5°, 45°, 67.5°, and 90°) for the three projectiles. The angle of attack (α) is defined as the angle between the projectile axis and *z*-axis. For the convenience of measurement, the projectiles remain parallel to the *z*-axis and flies along the projectile axis, and the sensor plate rotates to make an angle β (α = β) with the *Z*-axis (as shown in Figure 1). The same trend exists for the voltage value: when the angle of attack decreases, the sensor signal strength increases, especially within distances from 1 cm to 40 cm. Compared to the sensitivity at 90°, the sensitivities at different attack angles are enhanced by 20.11% (22.5°), 11.97% (45°), and 7.50% (67.5°) for p1; 33.81% (22.5°), 16.22% (45°), and 10.7% (67.5°) for p2; 22.21% (22.5°), 17.51% (45°), and 8.47% (67.5°) for p3. This trend can be explained by the polarization surface of the target increasing with the decrease of angle of attack.

## 5. Conclusions

In this paper, a moving-target proximity detection method using a planar capacitive sensor system is presented, and performance evaluation parameters for sensitivity distribution homogeneity have been discussed. To gain nice homogeneity in sensitivity distribution and high signal intensity, a long-scale (31 cm × 19 cm) sensor has to be designed. A planar capacitive sensor with a spiral shape is designed based on numerical simulations and experiments. A measuring circuit with reduced response time is designed. From the analysis of voltage variation curves of static experiments and dynamic experiments, it is clearly demonstrated that the PCSS can detect moving targets and is capable of capturing the target up to 60 cm away. This study suggests that the sensitivity of the proposed method is a feasible, inexpensive moving target detecting method for an interception system. Based on this method, further studies on detection of orientation, speed and distance of moving targets could be continued.

## Figures and Tables

**Figure 5:** Simulated electric potentials, electric fields and sensitivity maps of four electrode shapes: (**a**) Circular shape; (**b**) Rectangular shape; (**c**) Spiral shape and (**d**) Comb shape.

**Table:** 

	Electric potential	Electric field	Sensitivity map
**(a)**	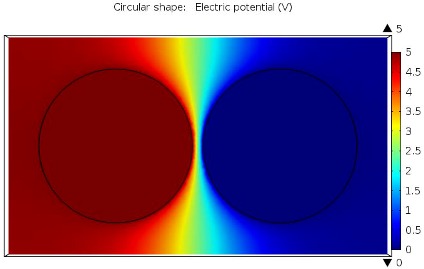	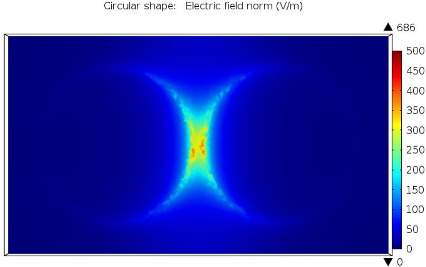	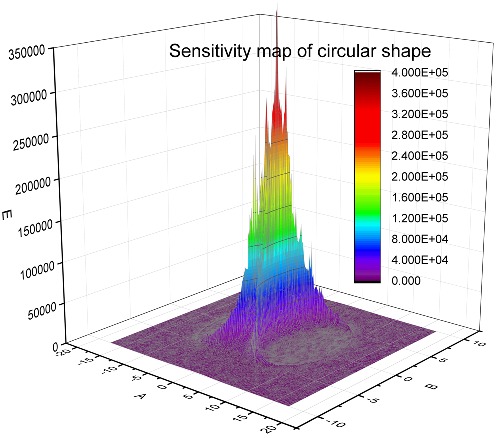
**(b)**	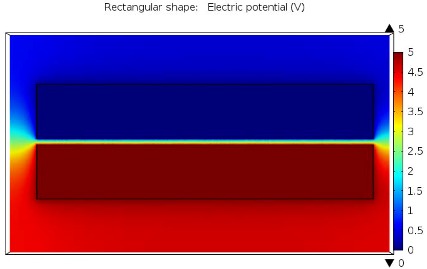	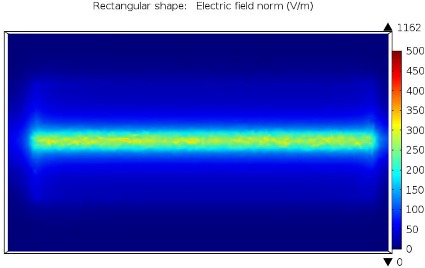	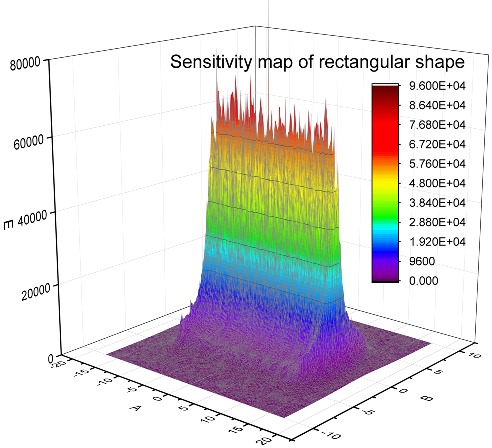
**(c)**	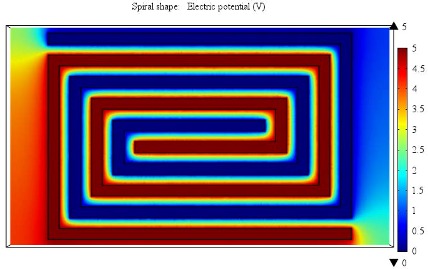	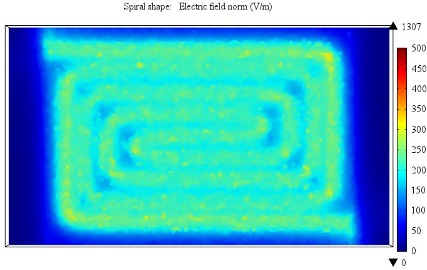	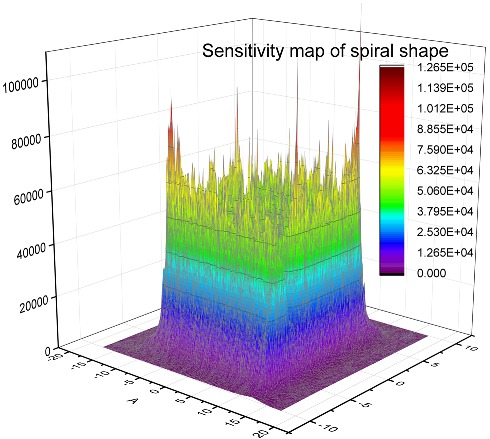
**(d)**	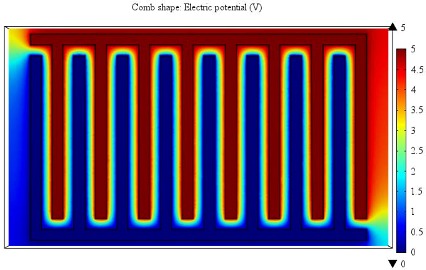	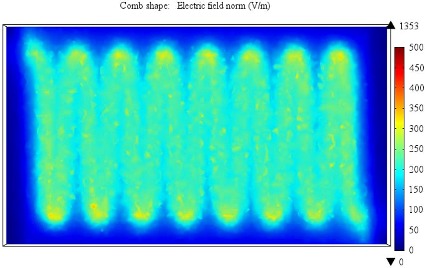	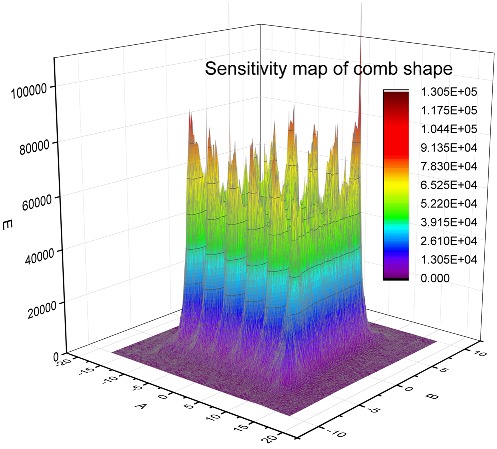

**Table 1:** The size of the three projectiles.

Projectile Structure	P1	P2	P3
Diameter/cm	5	2.8	1.4
Length of projectile body/cm	53	54	6.5
Length of warhead/cm	11	8	14
